# Study between anb angle and wits appraisal in cone beam computed tomography (cbct)

**DOI:** 10.4317/medoral.18919

**Published:** 2013-05-31

**Authors:** Natalia Zamora, Rosa Cibrián, Jose L. Gandia, Vanessa Paredes

**Affiliations:** 1 PhD, Orthodontist , Research Assistant Professor, Department of Orthodontics, Faculty of Medicine and Dentistry, University of Valencia, Valencia, Spain; 2 PhD, Associate Professor, Department of Physiology, Faculty of Medicine and Dentistry, University Valencia, Valencia, Spain; 3 PhD, Orthodontist, Professor and Department Chair of Postgraduate Orthodontics Masters Course, Department of Orthodontics, Faculty of Medicine and Dentistry, University of Valencia, Valencia, Spain; 4 PhD, Orthodontist and PhD Associate Professor, Department of Orthodontics, Faculty of Medicine and Dentistry, University of Valencia, Valencia, Spain

## Abstract

Objectives: To analyse the ANB and Wits values and to study correlations between those two measurements and other measurements in diagnosing the anteroposterior maxilo-mandibular relationship with CBCT. Study Design: Ninety patients who had previously a CBCT (i-CAT®) as a diagnostic register were selected. A 3D cephalometry was designed using one software package, InVivo5®. This cephalometry included 3 planes of reference, 3 angle measurements and 1 linear measurement. The means and standard deviations of the mean of each measurement were assessed. After that, a Pearson´s correlation coefficient has been performed to analyse the significance of each relationship. Results: When classifying the sample according to the anteroposterior relationship, the values obtained of ANB (Class I: 53%; Class II: 37%; Class III: 10%) and Wits (Class I: 35%; Class II: 56%; Class III: 9%) did not coincide, except for the Class III group. However, of the patients classified differently (Class I and Class II patients) by ANB and Wits, a high percentage of individuals (n=22; 49%), had a mesofacial pattern with a mandibular plane angle within normal values. A correlation has been found between ANB and Wits (r=0,262), occlusal plane angle and ANB (r=0,426), and mandibular plane angle and Wits (r=0,242). No correlation was found between either Wits or ANB in relation with the age of the individuals. Conclusions: ANB and Wits must be included in 3D cephalometric analyses as both are necessary to undertake a more accurate diagnosis of the maxillo-mandibular relationship of the patients.

** Key words:**Cone beam computed tomography, ANB, Wits, cephalometrics.

## Introduction

The orthodontic diagnosis of disharmonies between the skeletal bases of the cranium (1) is often undertaken by relating both the maxilla and the mandible to reference points located on the cranial base.

ANB was the cephalometric measurement most commonly used to describe the discrepancy between the skeletal bases (1). These angles logically depend on the landmarks that form them, so variations, for example, in the position of the Nasion due to growth, or due to a lack of accuracy when measuring it, can affect the relationship of the skeletal bases, affecting also de measurement of ANB (2).

Freeman (3) was one of the first authors who showed this fact, that alterations in the position of the Nasion could alter the value of the ANB angle. Later, other authors showed how the Nasion point change throughout growth adopting a more forward and upward position. They conclude, moreover, that point A was also not a fixed point, as it varied with growth in a similar way to the Nasion. Similar results were reported by Taylor (4), who stated that the ANB angle did not only depend on the Nasion, but also on facial divergence.

Oktay (1) summarised the factors that could affect the ANB angle as: the age of the patient (ANB decreases with age), the Nasion position, the rotation of the SN (Sella- Nasion) line, the occlusal plane, the maxillae and the facial prognathism.

In 1976, Jacobson (5) introduced the “Wits appraisal” (abbreviation of the University of Witwatersrand, Johannesburg. South Africa). This linear measurement analyses the antero-posterior relationship of the skeletal bases eliminating reference points on the cranial base (1). Jacobson (5) observed that when the maxilla and the mandible were related to cranial planes of reference, errors may arise as a consequence of variations in craniofacial physiognomy. Among these differences were the antero-posterior position of the Nasion with regard to the maxillae and the rotational effect of the maxillae in relation to the cranial structures of reference. Jacobson (5), explained that a high ANB in an individual with excellent occlusion could be caused by a forward position of the maxillae in relation to the Nasion and/or by a clockwise rotation of the maxillae with regard to the anterior cranial base. In these cases differences were observed between both measurements giving different values when using ANB or Wits. Furthermore, according to this author, the ANB angle was only reliable if the mandibular plane angle was normal. An increased mandibular plane would indicate a divergent pattern and, in many of these cases, an anterior cranial base with a higher inclination, which reduces the SNA angle and provides less reliable information. Likewise, the Wits value could be affected by the inclination of the occlusal plane (1,6).

Various authors have recommended the use of both, ANB and Wits, for diagnosing the antero-posterior discrepancies of the skeletal bases (3,6,7). Measuring values that refer to the antero-posterior relationship of the maxillae has been widely studied with conventional radiographic registers in 2D, but not in 3D using CBCT. This technology offers a more complete and accurate vision and measurement of all the craniofacial structures and cephalometric measurements.

Accuracy and reliability of cephalometric points has been widely studied (8-15) with CBCT, concluding the high accuracy of the CBCT systems in the spatial location of cephalometric points.

Landmarks, in 3D, are defined in the three planes of the space and can be adjusted in the different slices and views (14,15), increasing its reliability more so.

This technology allows clinicians to create new reference planes. Being the planes formed by three points, instead of two (as in records 2D), the accuracy of the measurements is much greater and opens the door to reassess all the measurements previously established (15).

Moreover, different studies have compared linear and angle measurements between lateral cranial radiographs (LCR) and 3D projections (16-19) obtained from the slices of a CBCT scanner. It has been shown no clinical significance difference, it thus being possible to use most of the values or “norms” established in 2D for 3D measurements. This means we can build on standards established to date to classify our patients. However, we will consider this new technology to further refine the accu-racy of the measurements.

The development and diffusion of this 3D cephalometry would be a breakthrough for the daily practice since it would not be necessary to transform data from a CBCT, with all the information that entails, to a 2D projection. The measurements will be made directly in the 3D reconstruction giving us a more realistic view of the structure that we face.

The aims of this study are to use the current 3D technology with CBCT to analyse the the ANB and Wits values and to study the correlations between those two measurements and among others when diagnosing the anteroposterior maxilo-mandibular relationship.

## Material and Methods

A study approved by the ethical committee of the Clinical University Hospital of the University of Valencia was undertaken.

The final sample was composed of 90 CBCTs of patients between the ages of 8 and 40 who had previously undergone a full cranial scan at the University of Valencia, Spain. The mean age was (18.05 years ± 8.69). The CBCTs used were obtained from the data base of those patients who had previously undergone such diagnostic tool because different reasons: included teeth like canines or third molars, agenesis or supernumerary teeth, all without moderate to severe skeletal asymmetries. No patient was scanned because of the purpose of the present study.

Each of the patients had undergone a scan using the i-CAT® (Imaging Sciences International, Hatfield, Pa) equipment. This CBCT device uses an amorphous silicon flat panel sensor to capture the fields of view (FOV). The FOV employed was the portrait mode that captures data in extended FOV mode and includes the full head of 170 mm in height x 230 mm in diameter with a scanning time of 8.9 seconds. It was set at medium quality and high resolution mode. It generates a total of 326 slices, with an image matrix size of 400x400. The voxel size is of 0.4 x 0.4 x 0.4 mm. The focal size is 0.5mm y and the size of its base is 119x142 cm. Tube voltage is 120 kV and its intensity 23.87 mAs. The size of the data files generated is in the order of 35 megabytes.

The raw data obtained from the CBCTs were imported to the InVivo5® software (Anatomage, San Jose, CA) which was used to visualize the slices and 3D images that are obtained from a CBCT. This is where the 3D reconstruction of the DICOM images (Digital Imaging and Communications in Medicine) is made.

Fourteen cephalometric points were defined on each of the three spatial planes (X, Y, Z) ( Table 1). The procedure for locating each point requires the selection of the most appropriate view (sagittal, coronal, axial) and then adjusting that point on the other views for better accuracy. Employing this location method, all the spatial positions of each point have been pinpointed on each of these axes as numerical values.

**Table 1:**
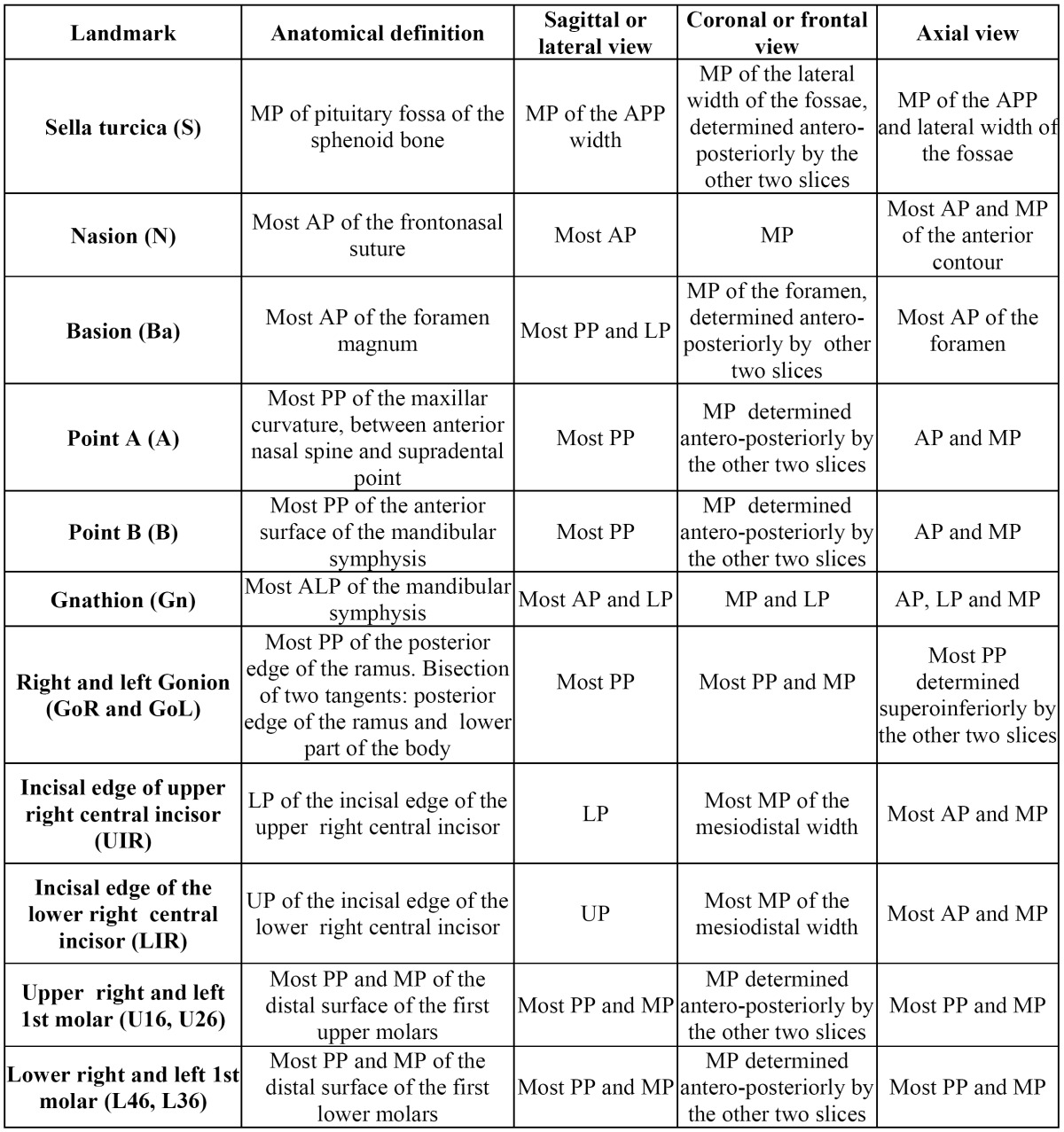
Definition of the three spatial planes of the 14 points used in this study. Anterior point (AP), anteroposterior point (APP), midpoint (MP), posterior point (PP), lowest point (LP), upper point (UP), antero-lower point (ALP).

In a previous study undertaken by the same authors (15), the reproducibility and reliability in locating cephalometric points was analyzed. To assess the reproducibility on landmark location, two observers with the same background and six years of experience in the field of orthodontics located 41 landmarks at three separate times. A total of 11,070 data were processed using the 15.0 SPSS statistical package®. To discover the reproducibility of the method on landmark location, an ANOVA analysis was undertaken using two factors of variation: time (t1, t2 and t3) and observers (Ob1 and Ob2) for each axis (X, Y and Z) and land-mark. The order of the CBCT scans submitted to the observers were different and randomly allocated. The intraclass correlation coefficient (ICC) was calculated. The results of the study showed that both intra-and inter-observer reliability were high, both being ICC ? 0.99.

Once the cephalometric points have been adapted to the 3D reality, that is, have been defined on each of the spatial planes (X, Y, Z) and have been duly located in their correct position, the next step was to design a 3D cephalometric analysis of the maxillo-mandibular relationship and the facial pattern.

**Figure 1:**
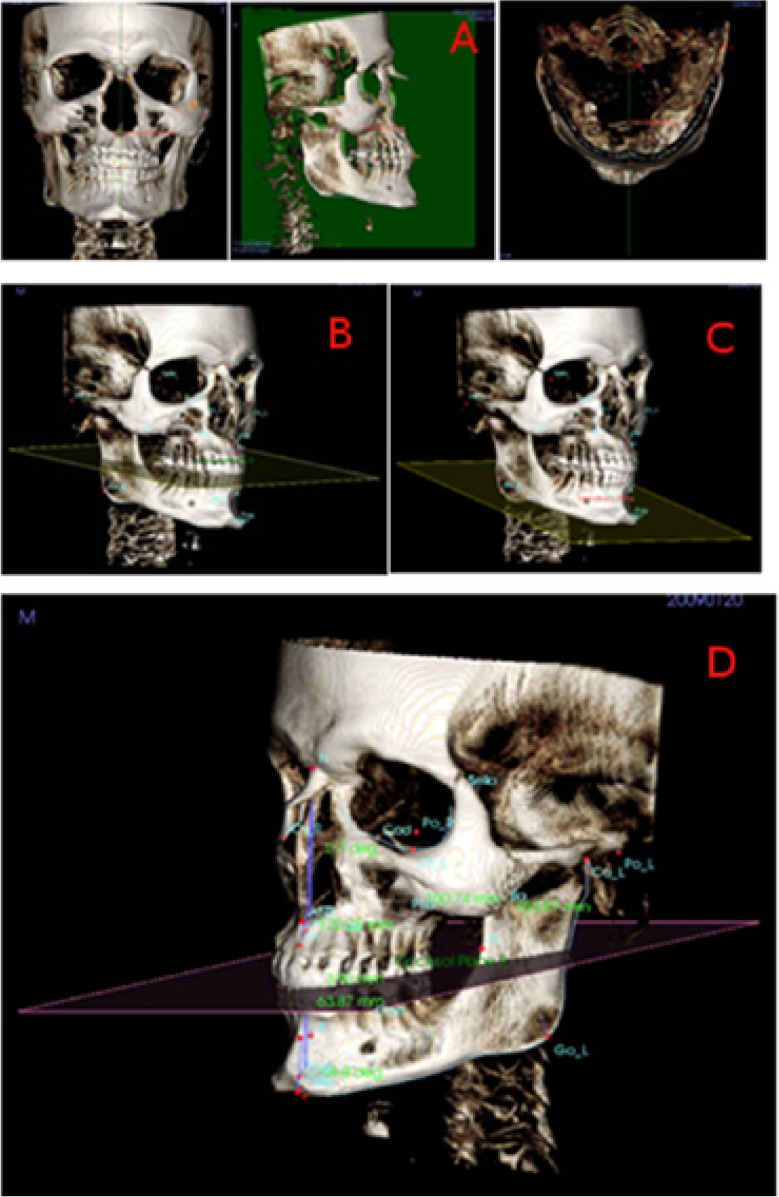
View of the mid-sagittal plane (coronal, sagittal, axial), the occlusal plane, the mandibular plane, and the measurements of skeletal class with the software InVivo5® (Anatomage, San Jose, CA).

First of all, three planes of reference have been described (Fig. 1).

1. Mid-sagittal plane (XZ) (Fig. 1): vertical plane of antero-posterior reference that divides the body in two (right and left por-tion), defined by the points N, S and Ba.

2. Occlusal plane (Fig. 1): plane that passes through the midpoint of the two upper and lower central incisors (URI-LRI), through the midpoint of the cuspids of the upper and lower molars of the right side and the midpoint of the cuspids of the upper and lower molars of the left side.

3. Mandibular plane (Fig. 1): plane that passes through the two Go (GoR- GoL) points and through the Gn point.

After that, 4 variables have been defined and analysed (Fig. 1):

4. ANB angle: angle formed by Nasion, A and B points. According to this measurement, the classification of the Angle Class would be distributed in the following way: Class I values of 2º± 2 (SD), Class II values >4º, Class III values <0º.

5. Wits Appraisal: distance between points A and B measured on the occlusal plane, as described by Jacobson (5). We have readapted this occlusal plane to the 3D reality, as described above. According to this measurement, the classification of the maloc-clusion would have the following distribution: Class I values of -1mm for men and 0mm for women, Class II values > -1mm for men and values > 0mm for women and Class III values <-1mm for men and values <0mm for women.

6. Occlusal plane angle: angle formed between the length of the anterior cranial base (SN) and the occlusal plane.

7. Mandibular plane angle: angle formed between the length of the anterior cranial base (SN) and the mandibular plane. Similarly to the occlusal plane, we have readapted this plane to the 3D reality, thus, adding more points to form the plane, as described above. According to Jacobson (5), mesofacial individuals would have values of 30º± 5 (SD) for men and 29.4º±6 (SD) for women. Over that value, individuals present a dolichofacial pattern and below it a brachyfacial pattern, as it is a good indicator of facial pattern.

An Excel® sheet, version 12.0 for Windows (Microsoft Corporation) was created to introduce all the variables and measurements corresponding to the three dimensional cephalometric analysis of the 90 patients. The data were introduced into version 17.0 of the statistical package SPSS® for Windows (SPSS Inc., Chicago, IL) for their subsequent analysis. The previously described measurements were selected and the means and standard deviations of the mean (SD) of each of them were found. In addition, the Pearson’s correlation coefficients were performed between the various variables described.

## Results

 Table 2, shows the overall mean values and standard deviation (SD) for the ANB angle, Wits Appraisal, occlusal plane and mandibular plane of all patients.

**Table 2:**
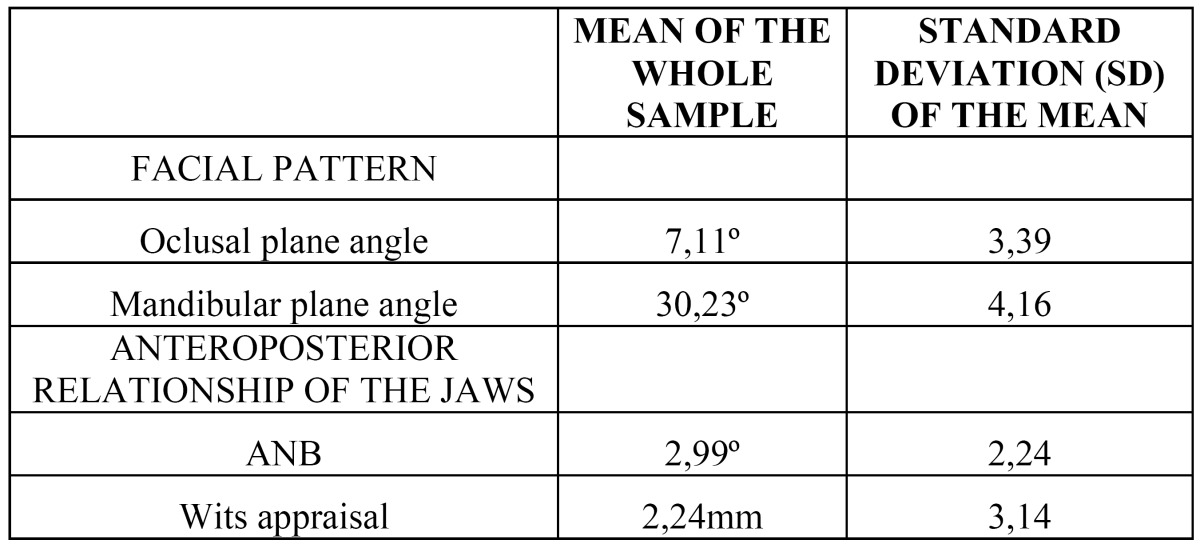
Mean of the whole sample and standard deviation of the mean (SD) of the measurements used in this study.

2 shows the percentages of anteroposterior relationships of the sample according to ANB and Wits Appraisal. According to the ANB angle, there would be 53% of patients with Class I, 37% with Class II and 10% with Class III, whereas according to Wits, there would be 35% of patients with Class I, 56% with Class II and 9% with Class III.

**Figure 2:**
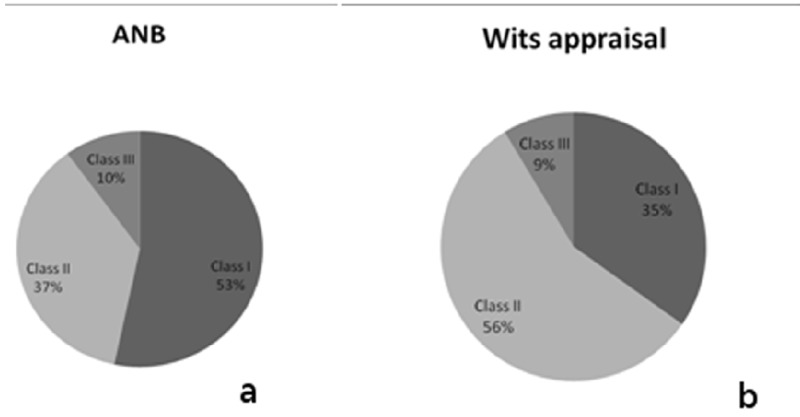
Sample classification according to antero-posterior skeletal values, ANB and Wits appraisal.

**Figure 3:**
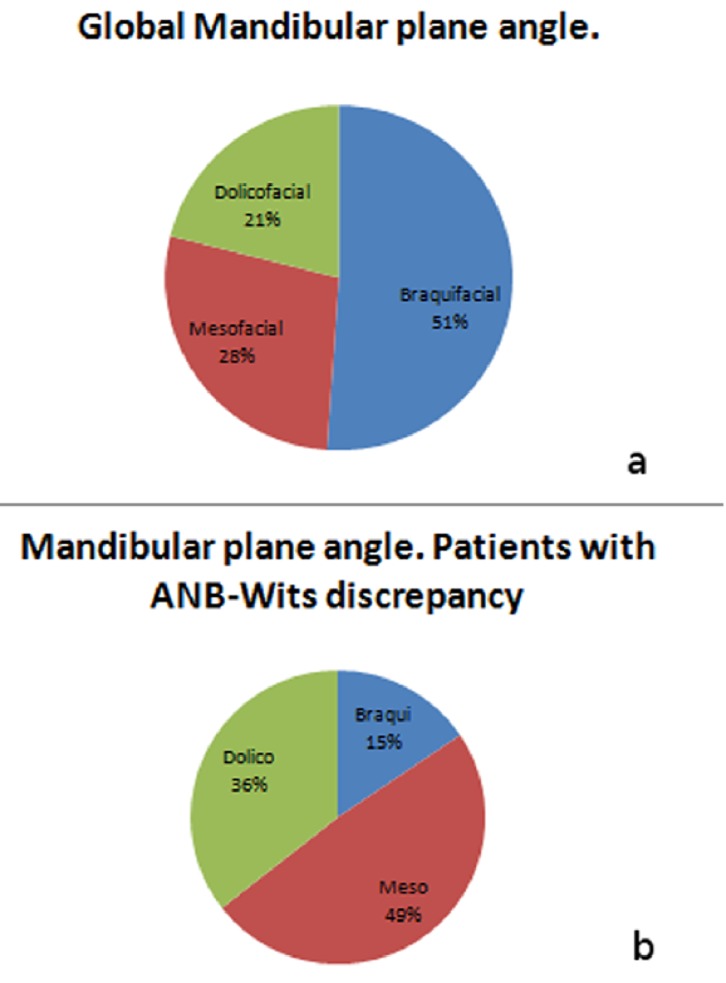
Sample classification according to mandibular plane angle. a) Distribution of the complete sample. b) Distribution of the 50% patients who present differences between ANB and Wits.

Another important aspect to be analysed was the facial pattern of the sample. Figure 3 shows the facial pattern of the whole sample, whereas (Fig. 3) shows the facial pattern of those patients who differ in their anteroposterior relationship depending on ANB and Wits. In figure 3 one can observe that 49% of the patients with ANB- Wits discrepancy have a mesofacial pattern, 32% a dolichofacial pattern and 19%, a brachyfacial pattern.

Results of the correlations between the different measurements are seen in  Table 3. A certain level of correlation between the oc-clusal plane angle with ANB, between the mandibular plane angle with Wits and between ANB with Wits may be observed. It has also been observed that there was no correlation of either ANB or Wits in relationship with the age of the sample.

**Table 3:**
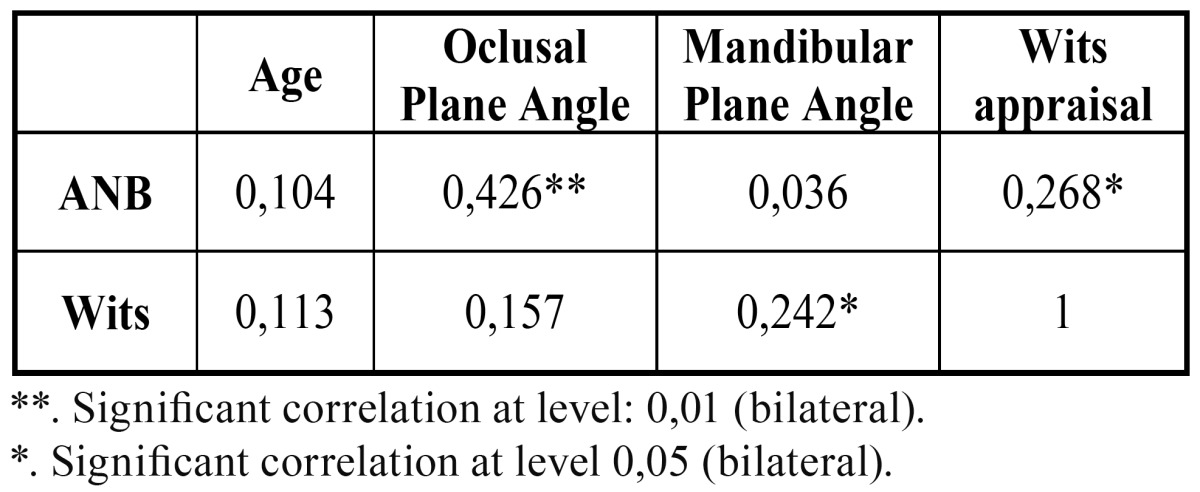
Correlation values.

## Discussion

To carry out this study, records of patients who had previously undergone a CBCT as a diagnostic tool because some additional alteration (agenesis, supernumerary teeth, included teeth…) were used; so undertaking a CBCT was justified. Patients with moderate and severe asymmetries were not included in this study.

In spite of the fact that there are already many studies on this issue, radiation that CBCT systems generate must continue to be carefully studied (20), as, to date, the radiation dose involved is considerably greater than that of conventional digital radiographs and their recommended use has to be fully justified (20).

Despite the fact that the sample was large, including 90 patients, the drawback of carrying out a study of these characteristics is that irradiating patients only for research purposes is not justified, creating in some cases difficulties to properly interpret the results.

Of the 90 patients analysed in our study, there was a difference between the ANB and Wits anteroposterior classification in 50% of the cases. ANB diagnosed more patients with Class I and fewer patients with Class II, and Wits diagnosed more patients with Class II and less with Class I. However, the number of Class III diagnosed was very similar for both ANB and Wits measurements.

Based on the results of this study, it can be seen how the anteroposterior relationship of our sample is classified differently depending on whether the ANB angle or Wits is used, especially for the class I and class II patients. These differences could appear because, as previously stated by several authors, the ANB angle is influenced by the anterior cranial base position and the possible rotation of the maxillae, whereas the Wits is influenced only by the occlusal plane, not having influence of the cranial base (5,7).

On analysing the facial pattern (based on the mandibular plane) of the 45 patients for whom the Wits and ANB diagnosis of anteroposterior relationship did not coincide, we observe that half of them, 22 out of 45 (49%), had a mesofacial pattern. This contrasts with the conclusions of Jacobson (5) who claimed that the ANB was only reliable if the mandibular plane was normal. This data indicates that, in our study, a high percentage of individuals, despite having a mesofacial pattern with a mandibular plane angle within normal values, present differences between ANB and Wits.

As regards the correlations found, our results show the highest one between occlusal plane angle and ANB (r=0,426). These results coincide with those of Hussels et al. (21) and Nanda (22). Moreover, these authors (21,22), like us, did not find any correlation between the mandibular plane angle and the ANB.

With regard to Wits appraisal, even though some studies (23) found a correlation of Wits with the occlusal plane angle, we did not find any. However, we did find a correlation between Wits and the mandibular plane angle.

We haven't find in our study any correlation of either Wits or ANB with regard to the age of the patients. Bishara et al. (7) found a correlation between ANB and age, but not with Wits. In his study, a reduction of ANB with age was observed. Other authors (22,24) have observed the same results as Bishara et al. (7) for ANB. The difference between our results and those of Bishara et al. (7) may be due to the sample selection and the way the study was undertaken, that authors (7) undertook a longitudinal study with multiple registers of each individual throughout their growth. This type of study is not possible to carry out nowadays with CBCT.

The results of our study show a slight correlation between ANB and Wits (r=0,268), results similar to those of Bishara et al. (7), although the values of correlation found by that author were slightly higher than ours.

Both Bishara et al. (7) results and ours have values of correlation coefficients less than r=0.8, meaning little predictive value when applied to an individual. Other authors (2,22) also claim that, given that ANB and Wits evaluate the same skeletal discrepancy, they must, in theory, have a high correlation. However, the fact is that the correlation between them is not as strong as expected, which suggests a certain weakness in at least one of the measures.

Due to the fact that the correlation between ANB and Wits is not as high as one would expect and to the fact that the position of the skeletal bases in relation to the anterior cranial base influences one measurement, but not the other, and that both measure-ments complement each other, both must also be included in 3D cephalometric analyses as both are necessary for undertaking, a more accurate diagnosis of the maxillo-mandibular relationship of the skeletal base and essential for individualizing each specific case.

As mentioned before (16-19), the angles and linear measurements employed in conventional 2D cephalometry could be extra-polated to 3D cephalometry. However, this 3D cephalometry provides us with a new dimension allowing us to create new planes of reference formed at least by three landmarks, and allowing us to evaluate characteristics that would be difficult to measure in 2D.

With the introduction of CBCT in orthodontic diagnosis, new possibilities have opened up in the study of cranio-facial relationships. These types of 3D analysis are going to be implemented in the future, for all the patients who need a CBCT. It is, thus, important to redefine measurements and reevaluate norms in order to diagnose the patients following also these criteria. That is why we considered of interest to create and study a sample of patients using these new technologies.

The conclusions of the present study are:

• There is no agreement between ANB and Wits in the diagnosis in 50% of the individuals of our sample.

• We have found slight correlation between ANB and Wits, a moderate correlation between the occlusal plane angle and ANB, a slight correlation between the mandibular plane angle and Wits and no correlation of either ANB or Wits with age.
